# An Obstruction of the Œsophagus Removed by a Tobacco Glyster, on the Third Day after the Accident

**Published:** 1807-01

**Authors:** W. Blair

**Affiliations:** Great Russell Street


### An Obstruction of the Œsophagus removed by a Tobacco Glyster, on the third Day after the Accident.

### To Dr. BRADLEY.

Dear Sir,

If the following recital should be the occasion of one person's life being saved, I am confident you will not think me troublesome in having called your attention to a fact of so simple a nature as the following, and to a mode of practice which might have appeared trifling in the event of an unsuccessful result. I do not think, however, that the most brilliant success of the most delicate manœuvre in Surgery, is to be compared with the relief afforded by a proposal so easy and obvious; for I had rather preserve life, by directing an *enema* to be administered, than by performing the rare operation of œsophagotomy.

An elderly woman applied to me yesterday, at the Bloomsbury Dispensary, quite incapable of swallowing any thing, either solid or liquid. This incapacity, she said, was caused by a morsel of beef having stuck fast in the act of taking her dinner three days before; but, at all times, she found some difficulty in swallowing solid food, perhaps from a partial stricture existing in the œsophagus. From this constriction, I apprehend, it was not in my power to pass a probang more than half way down towards the upper orifice of the stomach; so that, after several efforts, I could not afford her any relief by this means.

More than eight years ago,* I was consulted in a case nearly similar; when I ordered a drachm of tobacco to be infused, and employed as a glyster, with the view of exciting sickness. On that occasion, the intended effect was produced, and the patient was saved, by vomiting up the portion of meat. I now again had recourse to the same experiment, and had the pleasure to find the same happy result, on the administration of a second *enema*; for the first did not produce sickness, and therefore required a repetition, about half an hour afterwards.

*Great Russell Street,* *Nov.* 26, 1806. — I remain, &c. W. BLAIR.

---

* This case is described in the fifth volume of the Memoirs of the London Medical Society, Art. xxxiii. p. 328.

